# Supplementary material for: Efficacy and Mechanism of Polymerized Anthocyanin from Grape-Skin Extract on High-Fat-Diet-Induced Nonalcoholic Fatty Liver Disease
Source: Nutrients. 2019 Oct 27;11(11):2586. doi: 10.3390/nu11112586 (PMC6893447; doi:10.3390/nu11112586)
Supplement: Supplementary file 1 [file nutrients-11-02586-s001.zip › nutrients-583342-SI.pptx]

## Slide 1
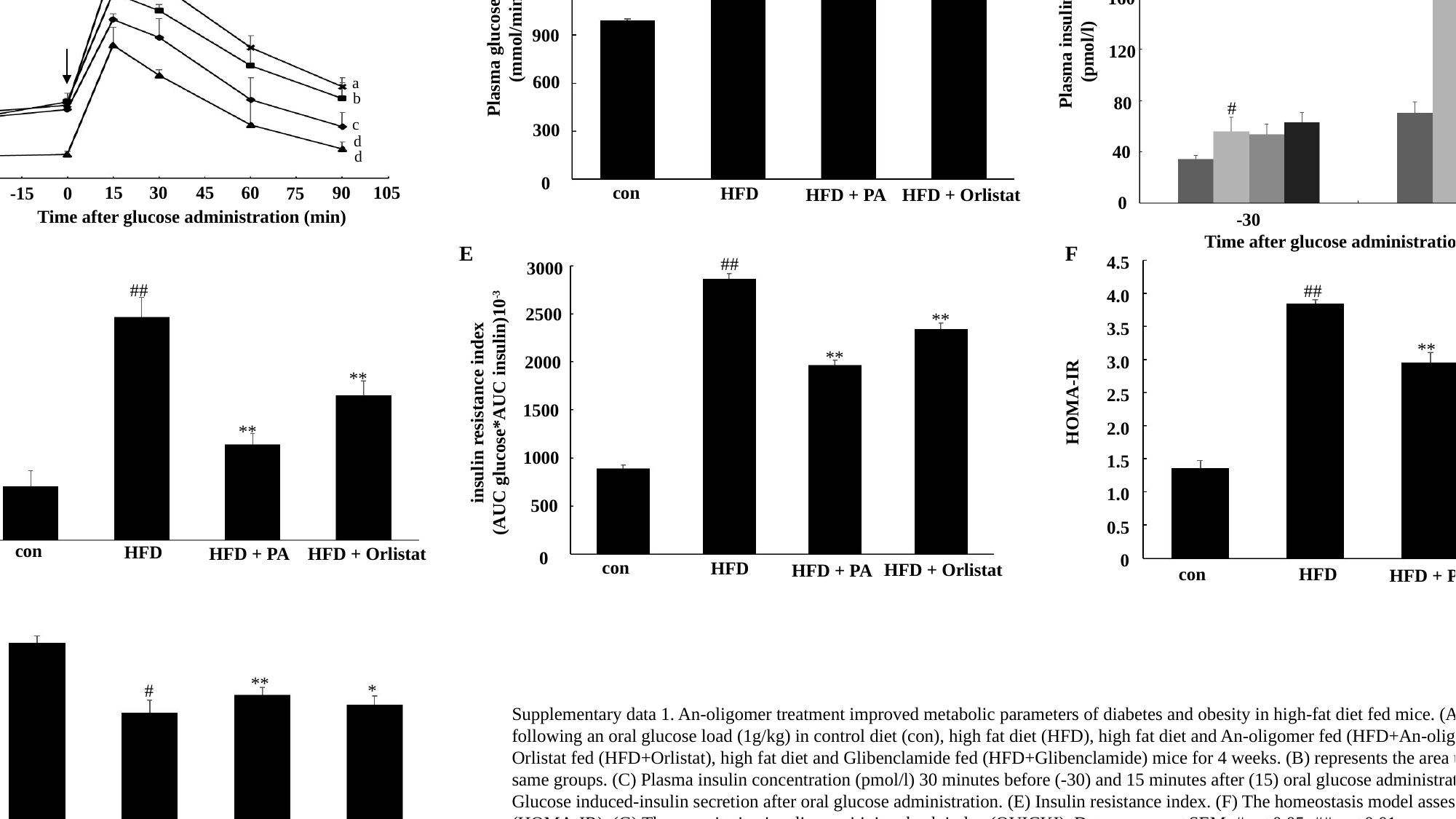

Metabolic parameters of diabetes and obesity
25
con
HFD
HFD + PA
Plasma glucose (mmol/l)
15
30
45
90
60
105
-30
-15
0
75
HFD + Orlistat
20
15
10
5
A
B
C
1800
1200
900
300
0
con
HFD
HFD + Orlistat
HFD + PA
Plasma glucose AUC
(mmol/min)
1500
600
##
*
*
240
con
HFD
HFD + PA
200
HFD + Orlistat
Plasma insulin (pmol/l)
160
120
80
40
0
15
-30
##
**
**
#
a
b
c
d
d
Time after glucose administration (min)
Time after glucose administration (min)
D
160
∆ insulin (pmol/l)
120
80
40
0
con
HFD
HFD + PA
HFD + Orlistat
##
**
**
F
E
4.5
##
4.0
*
3.5
**
3.0
2.5
HOMA-IR
2.0
1.5
1.0
0.5
0
con
HFD
HFD + Orlistat
HFD + PA
##
3000
2000
1500
500
0
con
HFD
HFD + Orlistat
HFD + PA
insulin resistance index
(AUC glucose*AUC insulin)10-3
2500
1000
**
**
0.6
0.5
**
*
#
0.4
QUICKI
0.3
0.2
0.1
0
con
HFD
HFD + Orlistat
HFD + PA
G
Supplementary data 1. An-oligomer treatment improved metabolic parameters of diabetes and obesity in high-fat diet fed mice. (A) Plasma glucose (mmol/l) following an oral glucose load (1g/kg) in control diet (con), high fat diet (HFD), high fat diet and An-oligomer fed (HFD+An-oligomer ), high fat diet and Orlistat fed (HFD+Orlistat), high fat diet and Glibenclamide fed (HFD+Glibenclamide) mice for 4 weeks. (B) represents the area under curve (AUC) of the same groups. (C) Plasma insulin concentration (pmol/l) 30 minutes before (-30) and 15 minutes after (15) oral glucose administration of the same groups. (D) Glucose induced-insulin secretion after oral glucose administration. (E) Insulin resistance index. (F) The homeostasis model assessment of insulin resistance (HOMA-IR). (G) The quantitative insulin sensitivity check index (QUICKI). Data are mean±SEM. #p < 0.05, ## p < 0.01 compared with the Con group. *p < 0.05, ** p < 0.01 compared with the HFD group. (n = 5 per group).
